# Antimicrobial and Anti-Inflammatory Activity of *N*-(2-Bromo-phenyl)-2-hydroxy-benzamide Derivatives and Their Inclusion Complexes

**DOI:** 10.3390/pharmaceutics17070869

**Published:** 2025-07-02

**Authors:** Ioana Maria Carmen Ienașcu, Adina Căta, Antonina Evelina Lazăr, Nick Samuel Țolea, Gerlinde Rusu, Paula Sfîrloagă, Cristina Moşoarcă, Adriana Aurelia Chiș, Claudiu Morgovan, Corina Danciu, Delia Muntean, Iuliana Popescu, Raluca Pop

**Affiliations:** 1 National Institute of Research and Development for Electrochemistry and Condensed Matter, 144 Dr. A. P. Podeanu, 300569 Timişoara, Romania; imcienascu@yahoo.com (I.M.C.I.); antonina_pop@yahoo.com (A.E.L.); samuelandnick@gmail.com (N.S.Ț.); paulasfirloaga@gmail.com (P.S.); m.crristina@gmail.com (C.M.); 2 Department of Pharmaceutical Sciences, Faculty of Pharmacy, “Vasile Goldiş” Western University of Arad, 86 Liviu Rebreanu, 310045 Arad, Romania; 3 Faculty of Industrial Chemistry and Environmental Engineering, Politehnica University of Timisoara, 6 Vasile Pârvan Bvd., 300223 Timisoara, Romania; gerlinde.rusu@upt.ro; 4 Association for Excellence in Pharmaceutical Education and Research, 25 Calea Cisnadiei, 550376 Sibiu, Romania; a.adriana.chis@gmail.com; 5 Preclinical Department, Faculty of Medicine, “Lucian Blaga” University, 2A Lucian Blaga Street, 550169 Sibiu, Romania; claudiu.morgovan@ulbsibiu.ro; 6 Faculty of Pharmacy, “Victor Babeș” University of Medicine and Pharmacy Timișoara, 2 Eftimie Murgu Square, 300041 Timișoara, Romania; corina.danciu@umft.ro (C.D.); muntean.delia@umft.ro (D.M.); pop.raluca@umft.ro (R.P.); 7 Research and Processing Center of Medicinal and Aromatic Plants, “Victor Babeș” University of Medicine and Pharmacy Timișoara, Eftimie Murgu Square, No. 2, 300041 Timişoara, Romania; 8 Faculty of Medicine, “Victor Babeș” University of Medicine and Pharmacy Timișoara, 2 Eftimie Murgu Square, 300041 Timișoara, Romania; 9 Multidisciplinary Research Center on Antimicrobial Resistance, “Victor Babeș” University of Medicine and Pharmacy Timișoara, 2 Eftimie Murgu Square, 300041 Timișoara, Romania; 10 Faculty of Agriculture, University of Life Sciences “King Mihai I” from Timisoara, 119 Calea Aradului, 300645 Timișoara, Romania

**Keywords:** ethyl ester, hydrazide, hydrazone, β-cyclodextrin, inclusion complex, antibacterial activity, anti-inflammatory activity

## Abstract

**Background/Objectives**: In order to enhance the biological activity, novel complexes of *N*-(2-bromo-phenyl)-2-hydroxy-benzamide derivatives and β-cyclodextrin were obtained. **Methods**: The inclusion complexes were characterized using spectral and thermal analyses. The antimicrobial activity was determined using the disk diffusion agar method, and completed with the minimum inhibitory concentration (MIC) values obtained by the broth microdilution method. The in vitro anti-inflammatory activity was evaluated using the protease inhibition assay. **Results**: The computed supramolecular architectures of the inclusion complexes showed that the most stable molecular arrangements correspond to the models in which the *N*-(2-bromo-phenyl)-2-hydroxy-benzamide derivatives are partially included in the cyclodextrin cavity. The antimicrobial screening showed that the compounds were active against Gram-positive bacteria (MIC = 2.5–5.0 mg/mL). Also, the evaluation of the proteinase inhibitory activity showed that the IC_50_ values of the title compounds (0.04–0.07 mg/mL) were much lower than that of the acetylsalicylic acid (0.4051 ± 0.0026 mg/mL) used as positive control, proving their superior efficiency in inhibiting trypsin activity. **Conclusions**: The complexation proved to be beneficial for both antimicrobial and anti-inflammatory effects.

## 1. Introduction

The rising incidence of microbial infections highlights the critical need to explore new antimicrobial therapies with specific target effects. While the management of these infections typically involves the use of various antimicrobial agents, it is widely acknowledged that the extensive use of these treatments is a primary factor contributing to the growing antibiotic resistance observed in bacteria [1]. For instance, an important Gram-positive pathogen, *Staphylococcus aureus*, is known for its capacity to develop methicillin resistance. Methicillin-resistant *S. aureus* (MRSA) has become one of the most challenging and persistent global health threats, responsible for severe nosocomial infections and, increasingly, for community-acquired infections associated with high morbidity and mortality [2,3].

Recognized by various international organizations, antibiotic resistance is now one of the most pressing challenges to human health. While the introduction of new antibiotics during the golden age of antimicrobial discovery helped to alleviate this issue, there are currently very few antibiotics in development [4].

Harmful stimuli, like pathogens, as well as irradiations and cell damage trigger the immune system to cause an inflammation response in the heart, pancreas, liver, kidney, lung, brain, intestinal tract and reproductive system, so tissue damage or disease could appear at these levels [5]. Currently used anti-inflammatory compounds with proven clinical efficacy, like aspirin, indomethacin, flufenamic acid, ibuprofen and so on, are generally acidic in nature and inhibit cyclooxygenase (COX) enzymes involved in prostaglandin synthesis in a non-selective manner. There are two main COX isoenzymes: COX-1, which is constitutively expressed and involved in physiological functions such as gastric mucosal protection, and COX-2, which is primarily induced during inflammation. Non-selective inhibition of both isoenzymes can lead to anti-inflammatory effects but also to gastrointestinal side effects, due to the suppression of COX-1 activity [6,7,8,9]. Due to the risks associated with the inflammation process, medicinal chemists are challenged to find more efficient anti-inflammatory agents.

Proteases delivered by pathogens are supposed to contribute to inflammatory-driven processes, some of them being precisely designed to help the microbes endure the host environment [10]. Thus, finding novel antibiotic molecules that also exert an inhibitory effect on proteinases, and have minimal side effects, is essential in order to correctly address the issue of infection treatment, and could also serve as a solution for the economic consequences of these diseases.

Salicylanilides have been widely researched because of their diverse biological activities, including in vitro antibacterial, antimicrobial, antifungal and antimycobacterial effects [11,12,13,14]. For example, *N*-(2-bromo-phenyl)-2-hydroxy-benzamide derivatives were tested against *Fusarium oxysporum*, *Sclerotinia sclerotiorum* and *Saccharomyces cerevisiae*, and the values of MIC ranged from 0.3 to 5.0 mg/mL [15]. Additionally, salicylanilides synthesized from methyl salicylate derived from gondopuro oil have demonstrated anti-inflammatory activity, evidenced by their ability to inhibit protein denaturation [16]. Moreover, some studies have shown that salicylanilides inhibited the epidermal growth factor receptor (EGFR) protein tyrosine kinases [17], and chloropyridinyl esters of salicylic acid have shown inhibition of the SARS-CoV-2 3CL protease [18]. In addition, hydrazides and their hydrazone derivatives exhibited an extensive range of biological effects, such as antioxidant, anti-inflammatory, anticonvulsant, analgesic, antiplatelet, cardioprotective, antidiabetic, anticancer, antimicrobial, antimycobacterial, antiprotozoal, antiparasitic, anti-HIV, and so on [19]. Thus, combining such pharmacophores could lead to the enhancement of the molecule’s biological activity.

Cyclodextrins can help alleviate the hydrophobicity and irritative effects of salicylanilides. Cyclodextrin inclusion complexes are commonly used in the food, cosmetics, and particularly the pharmaceutical industries, due to their drug delivery systems, which provide advantages such as improved bioavailability, solubility, stability, relatively low toxicity (high therapeutic index), enhanced pharmacokinetic properties, affordability, and the ability to mask undesirable tastes, thus improving patient compliance [20,21,22,23]. The formation of such complexes is influenced by hydrophobic interactions, the size of the guest molecules, and their properties, all of which play a role in the stability of the complexes. This process involves the partial or full encapsulation of a guest molecule, where a hydrophobic molecule, or a hydrophobic portion of a polar molecule, is inserted into the cyclodextrin (CD) cavity. This leads to the displacement of water molecules from the cavity, enhancing the aqueous solubility of the sample [24]. Thus, inclusion complexes of cyclodextrin can act as a drug release system which enhances the pharmacological profile of the entrapped substance [25].

Yet, the formation of inclusion complexes is not always guaranteed, and in some cases, the interaction may be weak or reversible, leading to suboptimal therapeutic performance [26,27,28]. Although CDs can modulate drug release, their inclusion complexes often suffer from rapid drug release, requiring further formulation advancements [28]. Native β-CD is common due to its cost and cavity size, but its low solubility and potential nephrotoxicity restrict parenteral use [29,30]. While CD inclusion complexes can improve the delivery of hydrophobic drugs like salicylanilides, their effectiveness varies and must be evaluated case by case. Further research is needed to optimize complexation and assess long-term safety and bioavailability.

**Figure 1:**
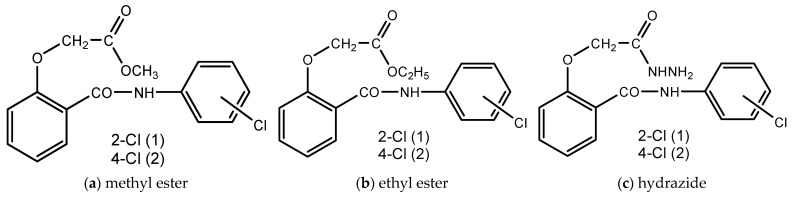
Chemical structures of (**a**) methyl ester, (**b**) ethyl ester and (**c**) hydrazide of *N*-(2-chlorophenyl)-2-hydroxybenzamide (1) and *N*-(4-chlorophenyl)-2-hydroxybenzamide (2).

Recently, our research team studied the antibacterial activity of novel *N*-(2-chlorophenyl)-2-hydroxybenzamide (1) and *N*-(4-chlorophenyl)-2-hydroxybenzamide (2) derivatives (Figure 1) and proved the beneficial role of complexation on the efficiency of these compounds. Thus, antimicrobial evaluation showed good activity on Gram-positive and no inhibition against Gram-negative bacteria, the most efficient compounds being the 2-chloro-substituted salicylanilide derivatives [23].

In order to enhance the studies in this field, the current research proposed the design of new 2-bromo-substituted salicylanilide derivatives, ethyl esters, hydrazides and hydrazones and their inclusion complexes in β-cyclodextrin, and the evaluation of the in vitro antimicrobial and anti-inflammatory activity of the novel molecules.

## 2. Materials and Methods

### 2.1. Chemicals

The *N*-(2-bromo-phenyl)-2-hydroxy-benzamide derivatives, [2-(2-bromophenylcarbamoyl)phenoxy]acetic acid ethyl ester (EE), *N*-(2-bromo-phenyl)-2-hydrazinocarbonylmethoxy-benzamide (HD), 2-(5-bromo-2-hydroxy-benzylidene-hydrazinocarbonylmethoxy)-*N*-(2-bromo-phenyl)-benzamide (HN) were obtained using microwave irradiation and characterized in previous research [15]. β-cyclodextrin (97%), trypsin solution from porcine pancreas (2.5 g/L), casein Hammarsten bovine (Sigma-Aldrich, St. Louis, MO, USA), trichloroacetic acid (99.5%) (Merck, Darmstadt, Germany); and absolute ethanol, and dimethyl sulfoxide (DMSO) solvents (Merck, Darmstadt, Germany, analytical purity) were used with no further purification.

### 2.2. Obtaining the Inclusion Complexes

The inclusion complexes (EEC, HDC, HNC) of the ethyl ester (EE), hydrazide (HD) and hydrazone (HN) in β-cyclodextrin (CD) were obtained using the kneading method [23]. The two components (1:1 molar ratio) were ground in an agate mortar for 30 min, with ethanol added to obtain a paste, which was held in a heating chamber at 50 °C for 5 days and stored in a desiccator.

### 2.3. Obtaining the Physical Mixtures

The physical mixtures (EEPM, HDPM, HNPM) of β-cyclodextrin (CD) and each of the *N*-(2-bromo-phenyl)-2-hydroxy-benzamide derivatives (EE, HD, HN) were prepared. First, the two components were individually grinded in a mortar. Then, both components, at a molar ratio of 1:1, were mixed together to form a unitary mixture [23].

### 2.4. Characterization Methods

FTIR spectra (KBr pellet, ν_max_ in cm^−1^) were acquired on a Vertex 70 spectrophotometer (Bruker Optik GmbH, Ettlingen, Germany) at a spectral resolution of 4 cm^−1^, 128 scans, room temperature, in the range 4000–400 cm^−1^. ^1^H-NMR spectra (DMSO-d_6_, 400 MHz) were recorded on an Avance DRX 400 instrument (Bruker, Billerica, MA, USA). Chemical shifts (δ values in ppm) are expressed aligned with tetramethylsilane (TMS). Coupling constants (*J*) are given in Hz. X-ray powder diffraction (XRD) data were collected using an X’Pert Pro MPD-type diffractometer (PANalytical, Almelo, The Netherlands) with Cu-Kα radiation (λ = 1.54060 Å) in the 2θ range of 5–80°. Scanning electron microscopy (SEM) analysis was performed using an Inspect S microscope (FEI, Eindhoven, The Netherlands), at 25–30 kV, in vacuum mode, at 400–12,000 magnification for all the samples. Thermogravimetric analyses (TGA) were carried out on a TG 209F1 Libra apparatus (Netzsch, Selb, Germany), in open alumina crucibles, under dynamic conditions, in an inert medium, N_2_, 20 mL/min., with a heating rate of 10 K/min, in the temperature range 25–500 °C. Differential scanning calorimetry analyses (DSC) were performed on a NETZSCH DSC 204F1 Phoenix apparatus (Netzsch, Selb, Germany), in an aluminum crucible with pierced lid, in an inert medium, N_2_, 20 mL/min., with a heating rate of 10 K/min, in the temperature range 25–350 °C. A Jasco V 530 UV-Vis spectrophotometer (ABL&E-JASCO, Vienna, Austria) was used for spectrophotometric measurements.

### 2.5. Determination of the Supramolecular Architecture

The three structures of the *N*-(2-bromo-phenyl)-2-hydroxy-benzamide derivatives were optimized at B3LYP/6-311+G level of theory, implemented in Gaussian 09W softwar, Revision D.01 [31]. The graphical representation of the frontier molecular orbitals HOMO and LUMO has been performed by means of GaussView 5.0 software. The partition coefficient logP, polarizability, polar surface area and steric parameters like ovality, molecular area and volume [32] were computed using pkCSM software (a freely accessible web server, available at https://biosig.lab.uq.edu.au/pkcsm/, last accessed on 29 May 2025) [33]. The molecular docking computations have been performed with AutoDock Vina version 1.5.6 [34]; the same software has been employed for the visualization of the ligand–receptor interactions. β-cyclodextrin was assigned as the receptor, and a grid box of 40 × 40 × 40 Å was employed, the center of the grid box being considered the center of the cyclodextrin.

The structure of the β-cyclodextrin (CHEBI 495055) was downloaded from ChEBI (Chemical Entities of Biological Interest) website [35]. Single-point energy calculation was performed for the inclusion complexes of β-cyclodextrin with the best conformation of the EE, HD, and HN ligands, at the B3LYP/6-31G level of theory implemented in Gaussian 09 software. Also, single-point calculations were performed for the corresponding ligands EE, HD, and HN and β-cyclodextrin from each of the three considered inclusion complexes. The results were employed for calculating the variation in energy associated with the formation of the β-cyclodextrin–ligand inclusion complexes.

### 2.6. Antimycobacterial Activity Evaluation

#### 2.6.1. Test Compounds

The three *N*-(2-bromo-phenyl)-2-hydroxy-benzamide derivatives and their inclusion complexes were evaluated for their antimicrobial properties. Three serial dilutions in DMSO of test compounds, from 10 to 2.5 mg/mL, were tested on microbial suspensions.

#### 2.6.2. Bacterial Strains and Antimicrobial Susceptibility Screening

For antimicrobial susceptibility test four Gram-positive and Gram-negative bacteria from American Type Culture Collection were selected—*Staphylococcus aureus* (ATCC 25923), *Streptococcus pneumoniae* (ATCC 49619), *Escherichia coli* (ATCC 25922) and *Pseudomonas aeruginosa* (ATCC 27853) (Microbiologics, Enghien-les-Bains, France).

According to the Clinical and Laboratory Standards Institute (CLSI), the antibacterial activity of the compounds was performed by the disk diffusion agar method [36]. All bacterial cultures were adjusted with NaCl 0.85% (bioMérieux, Marcy-l’Étoile, France) to a concentration of 0.5 McFarland, meaning 10^8^ CFU/mL (colony forming units). A blank paper disk (BioMaxima, Lublin, Poland) was placed on the surface of the agar Mueller–Hinton (MH) with 5% sheep blood (ThermoScientific, Dreieich, Germany) that was previously inoculated with the microbial suspension, and 10 µL of the test compound were added. The plates were incubated for 24 h at 35 °C. Then the inhibition zones were measured in millimeters using a ruler. Levofloxacin 5 µg and Gentamicin 10 µg disks (Oxoid, Altrincham, UK) were used as positive controls.

#### 2.6.3. Determination of Minimum Inhibitory Concentration (MIC)

The minimum inhibitory concentration (MIC) of compounds was determined using the broth microdilution method according to the CLSI guidelines [36]. The microbial suspension was prepared using a dilution of the standardized suspension (0.5 McFarland) to yield 5 × 10^5^ CFU/mL. Then, 500 µL of each test compound dilution, 250 µL Mueller–Hinton broth (bioMérieux, Marcy-l’Etoile, France) and 250 µL bacterial suspension were mixed, so the final tested concentrations were 5, 2.5 and 1.25 mg/mL. After 24 h of incubation at 35 °C, the lowest concentration without visible growth was considered the MIC.

The negative control consisted of 500 µL of DMSO, 250 µL broth and 250 µL bacterial suspension.

### 2.7. Evaluation of the Antiproteolytic Activity

Proteinase inhibitory activity of hydroxibenzamide derivatives was evaluated according to the method of Bijina et al. [37], with some adjustments. First, stock solutions (1.0 mg/mL) of the compounds in DMSO and additional dilutions in PBS (0.1−0.025 mg/mL) were prepared and used as test samples. The initial mixture (6 mL) consisted of 0.1 mL of trypsin, 4.9 mL of 0.1 M phosphate-buffered saline (PBS, pH 7.6), and 1 mL of the test sample was incubated (37 °C for 10 min), and then 2 mL of 0.75% (*w*/*v*) casein in PBS was added, and the mixture was further incubated (37 °C for 20 min). After cooling at room temperature, the reaction was terminated by the addition of 2 mL of 10% trichloroacetic acid (TCA) solution. The final mixture (10 mL) was centrifugated at 3000 rpm for 15 min (Hettich EBA 20 centrifuge, Hettich GmbH, Tuttlingen, Germany). The absorbance of the clear supernatant was measured at 210 nm against appropriate blanks (all mixture components except casein, subjected to identical incubation conditions), after appropriate dilution to ensure that absorbance values remained below 2.0.

The inhibition percentage of trypsin was calculated using Formula (1):

% inhibition of trypsin = 100 (1 − A2/A1),(1)

where A1 = absorption of the control sample (all mixture components except test sample), and A2 = absorption of the test sample.

The values of the IC_50_ were graphically obtained from the obtained linear regressions.

## 3. Results and Discussion

To enhance the pharmacotechnical and pharmacological properties of hydroxybenzamide derivatives, these compounds were incorporated into β-cyclodextrin, a naturally occurring and cost-effective cyclodextrin, whose hydrophobic cavity is well-suited for the encapsulation of such molecules.

**Figure 2:**
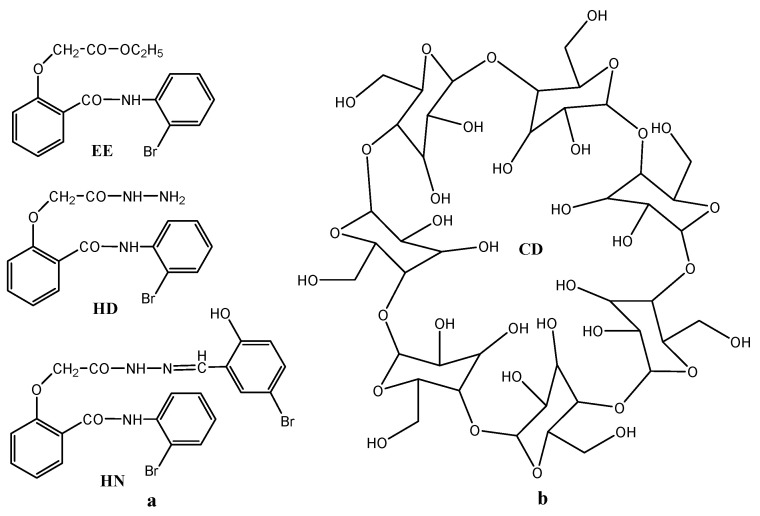
Structures of the two components of the inclusion complexes: (**a**) guests; (**b**) host.

Thus, the components of the studied inclusion complexes are, on the one hand, β-cyclodextrin (CD) and on the other hand, the molecule of [2-(2-bromophenylcarbamoyl)phenoxy]acetic acid ethyl ester (EE), *N*-(2-bromo-phenyl)-2-hydrazinocarbonylmethoxy-benzamide (HD) and 2-(5-bromo-2-hydroxy-benzylidene-hydrazinocarbonylmethoxy)-*N*-(2-bromo-phenyl)-benzamide (HN), respectively. The structures implied in the formation of the inclusion complexes are presented in Figure 2a (guests) and Figure 2b (host).

### 3.1. Molecular Modeling

The HOMO–LUMO gap, the energy difference between the frontier molecular orbitals, was employed for the evaluation of the stability of the compounds.

A previous study by our research group [38] dealt with the investigation of the frontier molecular energies of the ester by using a different functional (BLYP, a local density approximation functional). For the present investigation, which involves the comparison among the three investigated compounds, a more accurate prediction of the molecular structures and properties is needed. Therefore, the more accurate hybrid functional B3LYP [39] was chosen over the one previously employed.

The calculated results, together with the energies of the HOMO and LUMO orbitals, are given in Table 1:

The results suggest a higher stability for both the ester and hydrazide, compared to the results calculated for hydrazone. For the latter compound, a decrease in the HOMO energy and an increase in the LUMO energy were obtained.

**Figure 3:**
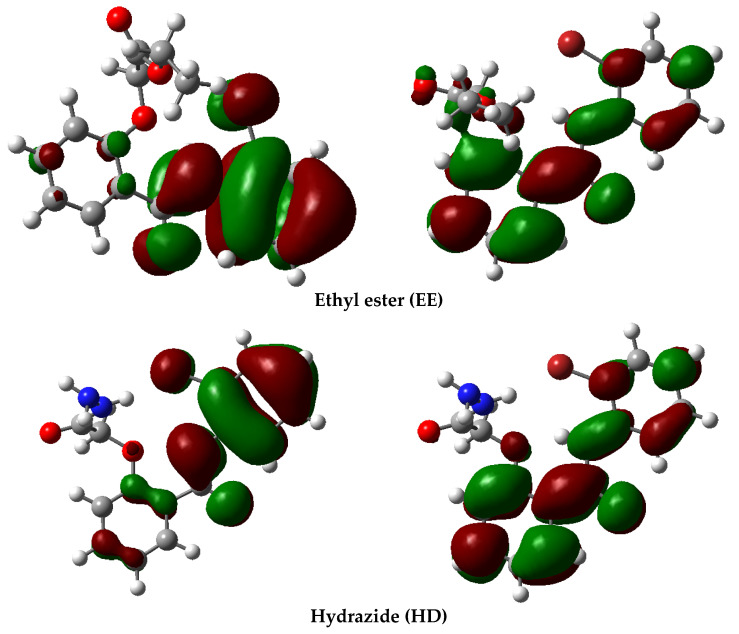

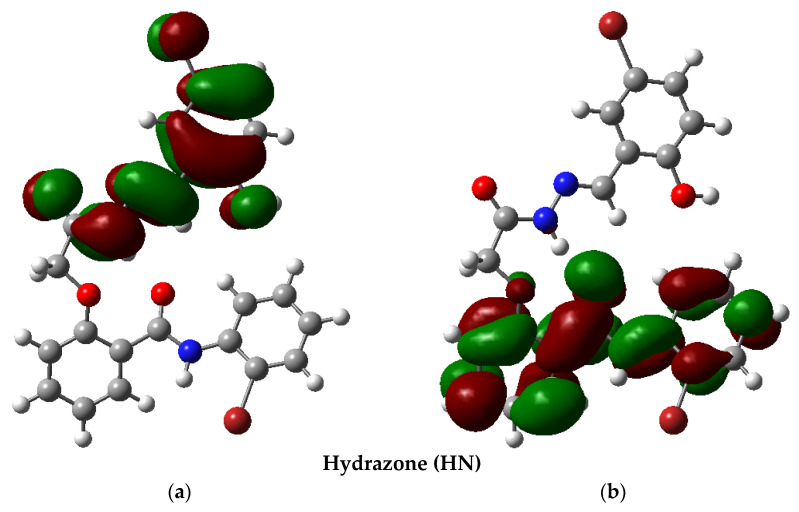
Graphic representation of frontier molecular orbitals of the ethyl ester (EE), hydrazide (HD) and hydrazone (HN): (**a**) HOMO orbitals; (**b**) LUMO orbitals.

A graphic representation of the frontier molecular orbitals is depicted in Figure 3:

The results depicted in Figure 3 outline the similarities between the ester and hydrazide; for both compounds, the HOMO orbitals are located on the 2-bromo-phenyl moiety, while the LUMO orbitals are delocalized over the two aromatic cycles. Concerning the hydrazone, the main difference appears at the HOMO orbitals; they are present over the benzaldehyde moiety, not on the 2-bromo-phenyl residue. Also, the LUMO orbitals of the hydrazone are located on the two aromatic rings of the salicylanilide structure.

The results presented in Table 2 outline the similarities of properties like ovality, molecular area and volume for the ester and the hydrazide. Regarding the calculated values of the polar surface area, larger values were obtained for hydrazide and hydrazone, compounds with free amine and hydroxyl groups. According to the results in Table 3, all the investigated compounds are hydrophobic (a larger logP value being obtained for hydrazone, a compound with three phenyl rings).

**Figure 4:**
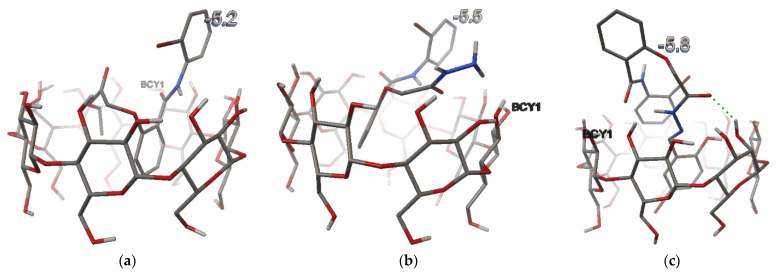
Structures of β-cyclodextrin inclusion complexes with: (**a**) ethyl ester (EE); (**b**) hydrazide (HD); (**c**) hydrazone (HN).

The results of the molecular docking study are included in Table 4:

The size of β-cyclodextrin cavity allows the partial inclusion of the *N*-(2-bromo-phenyl)-2-hydroxy-benzamide derivatives (Figure 4). As shown in Table 4, the best binding affinities were obtained for hydrazone (−5.59 kcal/mol).

The interactions of the *N*-(2-bromo-phenyl)-2-hydroxy-benzamide derivatives with β-cyclodextrin are shown in Table 5.

Regarding the interaction between the *N*-(2-bromo-phenyl)-2-hydroxy-benzamide derivatives and β-cyclodextrin (Table 5), in all three cases, atoms in close contact were present, and only in case of hydrazone (HN), one hydrogen bond is formed. These results suggest the partial inclusion of the compounds within the β-CD cavity.

**Figure 5:**
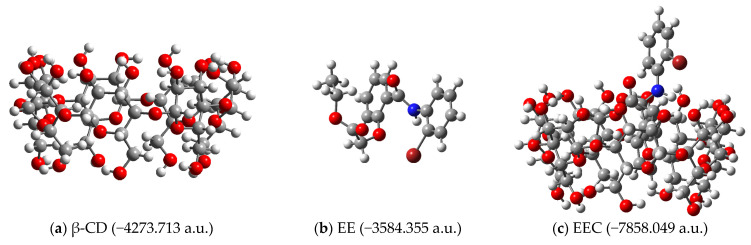
Structures of (**a**) receptor (β-CD); (**b**) ligand ethyl ester (EE); (**c**) inclusion complex (EEC), as obtained by molecular docking (B3LYP/6-31G).

**Figure 6:**
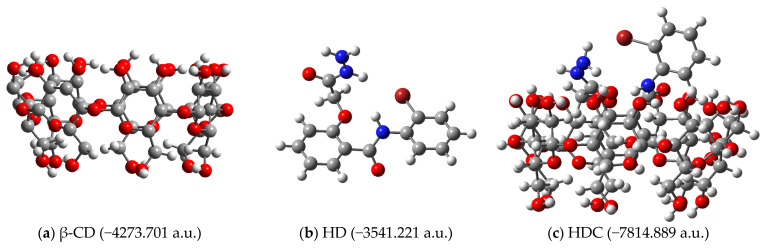
Structures of (**a**) receptor (β-CD); (**b**) ligand hydrazide (HD); (**c**) inclusion complex (HDC), as obtained by molecular docking (B3LYP/6-31G).

**Figure 7:**
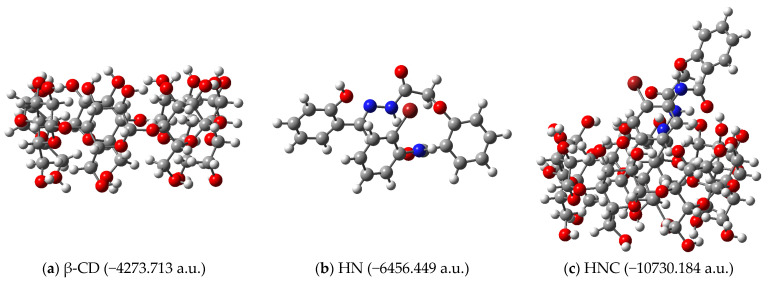
Structures of (**a**) receptor (β-CD); (**b**) ligand hydrazone (HN); (**c**) inclusion complex (HNC), as obtained by molecular docking (B3LYP/6-31G).

The complexation energies associated with obtaining the inclusion complexes depicted in Figure 4 were calculated. The structures of the receptors, ligands and complexes (together with their calculated energies) are depicted in Figure 5 for ethyl ester EE as ligand, Figure 6 for hydrazide HD as ligand and Figure 7 for hydrazine HN as ligand, respectively.

The results presented in Table 6 suggest the endothermic process for the obtaining of the inclusion complexes of ethyl ester EE and hydrazide HD, and an exothermic one for the hydrazine HN complex with β-cyclodextrin.

Molecular docking computations also suggested that hydrazine HN, characterized by the largest area, volume and polarizability within the series, has the highest binding affinity towards β-cyclodextrin.

### 3.2. Spectral and Thermal Characterization

In order to compare the results obtained for the inclusion complexes, the physical mixtures containing the two components of the complex were also prepared and analyzed. Characterization measurements were performed using the following modern analytical methods: X-ray diffraction, SEM, FTIR, ^1^H-NMR, TGA and DSC.

The following abbreviations were used to express the obtained results: EE—ethyl ester; CD—β-cyclodextrin; EEPM—physical mixture of CD and EE; EEC—inclusion complex of CD and EE; HD—hydrazide; HDPM—physical mixture of CD and HD; HDC—inclusion complex of CD and HD; HN—hydrazone; HNPM—physical mixture of CD and HN; HNC—inclusion complex of CD and HN.

**Figure 8:**
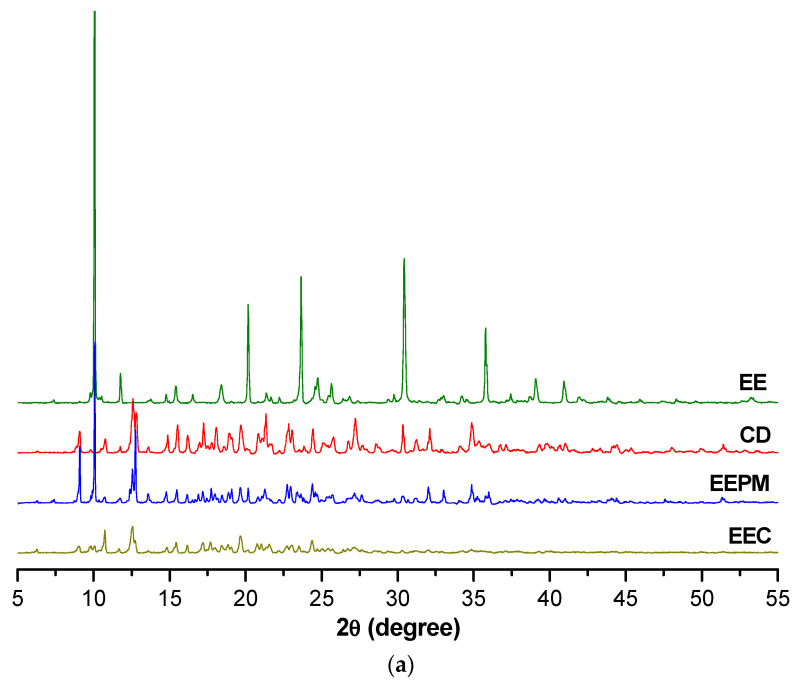

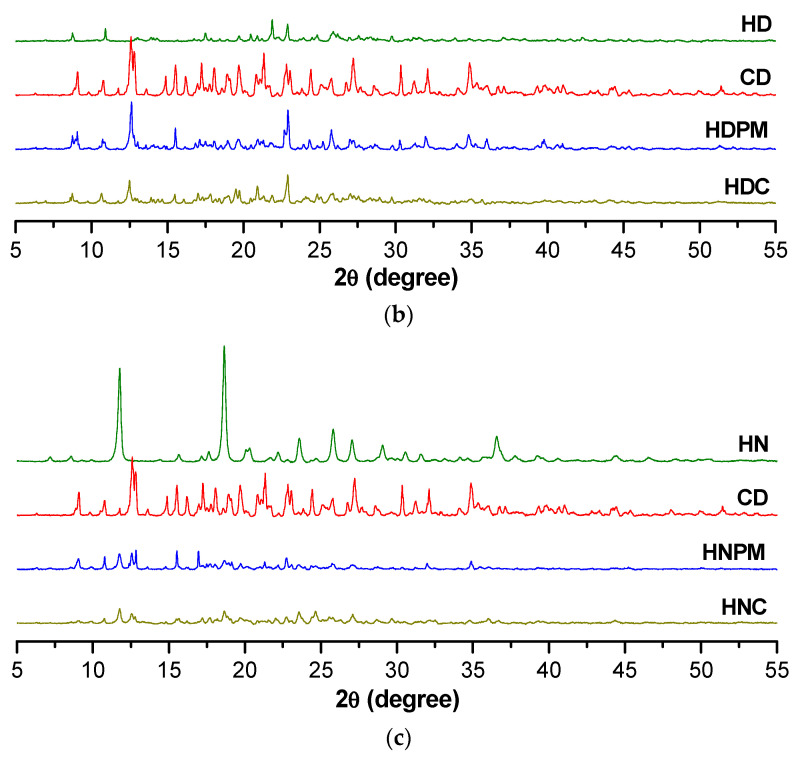
X-ray diffraction spectra: (**a**) ethyl ester set; (**b**) hydrazide set; (**c**) hydrazone set.

Figure 8 presents the X-ray diffraction spectra of the three analyzed sets corresponding to the ethyl ester, hydrazide and hydrazone series, along with the β-cyclodextrin, their physical mixtures and inclusion complexes.

The XRD patterns of the physical mixture slightly differ from the pure mathematical sum of its components. This could be due to the microstructural changes, partial amorphization and weak interactions during mixing, even if a true inclusion complex has not yet formed [40,41,42].

The analysis of the compounds by X-ray diffraction indicates, in the case of the binary compounds, the decrease in the crystallinity degree in comparison with the guest substances (EE, HD, HN) due to the amorphization phenomenon, and also changes in the position and intensity of the diffraction peaks. These alterations prove the formation of new solid states and demonstrate the presence of interactions between the hydroxybenzamide compounds and the cyclodextrin.

**Figure 9:**
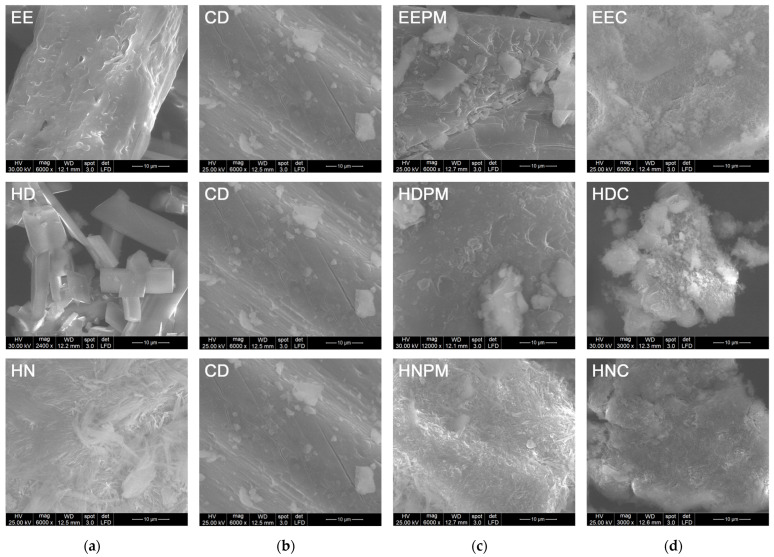
SEM images: (**a**) guests; (**b**) host; (**c**) physical mixtures; (**d**) inclusion complexes.

In order to evaluate the surface morphology and the dimension of the crystals, the SEM images of the *N*-(2-bromo-phenyl)-2-hydroxy-benzamide derivatives, β-cyclodextrin, their physical mixtures and inclusion complexes were recorded (Figure 9).

Regarding the physical mixtures (Figure 9c), there are no major differences between these and the cyclodextrin sample (Figure 9b), only in the case of the HNPM, hydrazone crystals can be better distinguished. However, the crystals are more agglomerated in the PMs compared to the inclusion complexes. For the inclusion complexes (Figure 9d), amorphous aggregates with asymmetrical shapes were observed, with a certain uniformity of the obtained precipitated particles. The dimensions of the particles are in the range of hundreds of nanometers, which is better observed at higher magnification.

**Figure 10:**
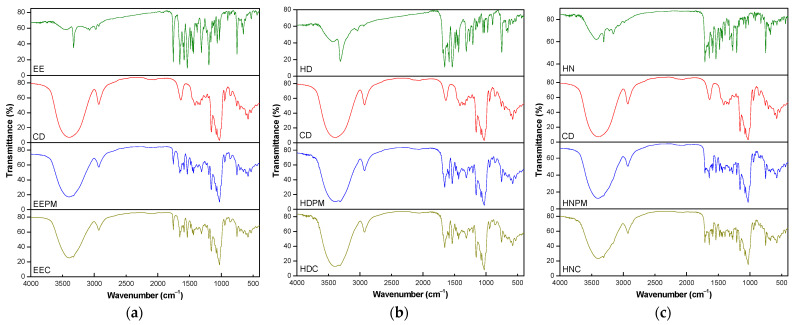
FTIR spectra: (**a**) ethyl ester set; (**b**) hydrazide set; (**c**) hydrazone set.

FTIR is an important analysis to demonstrate the existence of host–guest interaction in the molecule of inclusion complexes. FTIR spectra of the guest, β-cyclodextrin and their mixtures are presented in Figure 10.

Some characteristics of the IR spectra of the inclusion complexes (EEC, HDC, HNC), also observed by other researchers and important to prove the complex’s formation [43,44,45], are the similarity between spectra of the complexes and the CD spectra, and also the narrowing of the absorption band (3400.36 cm^−1^) corresponding to the hydroxyl groups of pure CD in the spectra of complexes. The IR spectra of the physical mixtures are a combination of the spectra of the neat compounds, where the characteristic bands of the guest and the cyclodextrin stand out.

Table 7 and Table 8 show minor differences between the spectra of the inclusion complexes and the spectra of the components (guests and host). Even small, the changes in the characteristic vibrations of the guests and host after complexation may suggest the existence of interactions between the components of the complexes and the partial entrapment of the guest within the hydrophobic cavity of β-cyclodextrin. However, the IR shifts must be correlated with other data sets, such as ^1^H-NMR, as the other technique provides additional evidence to support the significance of those changes.

The observed changes in the IR spectra of the complexes consist in minor shifts in certain bands frequency to higher or lower values, and also the modification of certain bands intensity. For example, in the case of the ethyl ester and hydrazide, the frequency corresponding to N–H stretching vibrations (νNH) of secondary amide, slightly shifts to a higher value in the complexes (Table 7), which can be related to the location of the amide group inside the cyclodextrin cavity (Figure 4b). In addition, for hydrazone, the intensity of the carbonyl group (νC=O ester) increases in the complex compared with the physical mixture, probably due to the hydrogen bond [46] formed with the hydroxyl group of the cyclodextrin (Figure 10c), which is in agreement with the molecular modeling (Figure 4c). The ether linkage (COC aromatic), present in all three guests, seems to be more affected by complexation phenomena in case of the ethyl ester, being in accordance with the molecular docking data, showing the presence of ethyl ester molecule inside the CD cavity, only the bromophenyl part of the molecule remaining outside (Figure 4a).

The entrapment of the guests within the hydrophobic cavity of β-cyclodextrin is also suggested by the slight changes in the C–H stretching vibrations (ν[CH_2_]) of β-cyclodextrin, which are more evident in the hydrazone inclusion complex (Table 8). The insertion of the benzene core inside the cyclodextrin, consecutively with the increase in electron cloud density, results in the changes in frequency [47]. On the other hand, the alteration of the microclimate with the development of hydrogen bonds and van der Waals interactions lead to the decrease in some band frequency [48]. The absence of extra bands in the inclusion complexes’ spectra prove that the interactions between the complexes’ components are only of a physical nature.

**Figure 11:**
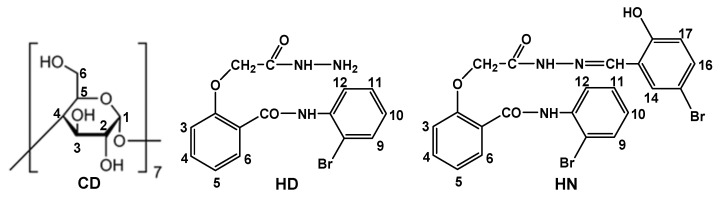
Protons number for host (CD) and guests (HD, HN).

^1^H-NMR, along with FTIR, represents a significant investigation to prove the formation of inclusion complexes. ^1^H-NMR data for the [2-(2-bromophenylcarbamoyl)phenoxy]acetic acid ethyl ester, β-cyclodextrin and their inclusion complex were presented in a previous paper [38]. ^1^H-NMR spectra of hydrazide, hydrazone, β-cyclodextrin and their complexes are presented in Figures S1–S5 (Supplementary File). Figure 11 shows the number of the host and guests protons used for the interpretation of NMR spectra.

The chemical shift changes (Δδ) between the free and complexed state of CD are presented in Table 9. Changes in the chemical shifts in the host and guest molecules, visible in the NMR spectra, are due to the insertion of the more hydrophobic part of the guest molecule in the hydrophobic cavity of the cyclodextrin. Thus, the most affected cyclodextrin protons should be the ones positioned inside the cavity of the cyclodextrin (H-3 and H-5) [49].

Table 9 reveals that the chemical shift changes corresponding to the protons located inside the β-cyclodextrin cavity are more evident than those manifested by the protons located outside the cavity, especially for the inclusion complex of hydrazone, demonstrating the formation of the inclusion complexes [49]. These findings are also supported by IR results, where the inclusion of the guests into the cavity of β-cyclodextrin was shown by the changes in the C-H stretching vibrations of β-cyclodextrin, and also more evident in the hydrazone inclusion complex (Table 8). Moreover, all computed supramolecular architectures of the studied complexes illustrate the partial inclusion of the guests into the β-cyclodextrin cavity and the presence of hydrophobic interactions inside the cyclodextrin cavity.

Table 10 and Table 11 present the chemical shift changes between the free and complexed hydrazide, for aromatic and non-aromatic protons, respectively.

The ^1^H-NMR results shown in Table 10 indicate slight modification of the chemical shifts corresponding to the aliphatic part of the hydrazide (∆δ_H_ ≤ ±0.005), the most affected by complexation seems to be the protons of OCH_2_CO group. Encapsulation at this level was also suggested by modification of the ether linkage frequency in the IR spectra of the hydrazide (Table 7) and supported by the molecular docking (Figure 4b). Regarding the chemical shift changes corresponding to non-aromatic protons, both the salicylic and benzanilide part of the hydrazide molecule seems to be influenced by complexation (∆δ_H-3_ = −0.004, ∆δ_H-6_ = −0.010, ∆δ_H-10_ = −0.007, ∆δ_H-12_ = −0.008), as shown in Table 11. The frequency shift in the N–H stretching vibrations observed in the IR spectra of the hydrazide complex (Table 7), corroborated NMR results and molecular modeling data (Figure 4b), attesting to the inclusion of salicylic nucleus inside the cyclodextrin cavity.

Table 12 and Table 13 present the chemical shift changes between the free and complexed hydrazone, for aromatic and non-aromatic protons, respectively.

Thus, regarding the hydrazone, the hydroxyl group (∆δ_OH_ = +0.009) attached on the benzaldehyde moiety and the -NH- group (∆δ_CONHN_ = +0.006) bonded to imine group seem to be affected by the inclusion into the cyclodextrin cavity (Table 12). These findings support the results obtained from IR spectra, where also slight modification of the N–H frequency was observed (Table 7).

Among the aromatic protons (Table 13), the most affected by complexation appear to be the H-10 (∆δ_H-10_ = −0.004), which is enclosed by the cyclodextrin OH groups, and H-17 (∆δ_H-17_ = −0.004), contained in the benzaldehyde moiety, that is included in the hydrophobic environment of the cyclodextrin cavity, based on the computational data regarding the ligand–receptor interactions (Figure 4c).

The NMR data for free and complexed hydrazide and hydrazone prove the hypothesis resulted from molecular modeling, namely, the partial encapsulation of the guest within the cyclodextrin cavity. This conclusion was also drawn in the case of ethyl ester [38].

**Figure 12:**
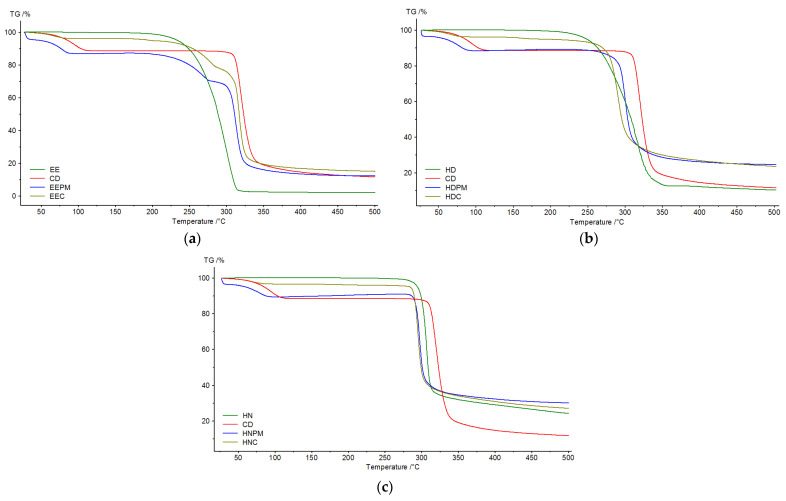
TGA curves: (**a**) ethyl ester set; (**b**) hydrazide set; (**c**) hydrazone set.

In Figure 12, the TGA curves of the guest, β-cyclodextrin, physical mixture and inclusion complex for the three series, EE, HD and HN, are plotted.

Thermogravimetric analysis was accomplished to detect the mass loss in relation to the temperature change. Thermograms were recorded for pure guests (EE, HD, HN), β-cyclodextrin, physical mixtures and inclusion complexes. The TG data, in the temperature range 25–500 °C, are depicted in Figure 12.

β-cyclodextrin displays two mass losses, one corresponds to the loss of water placed in the CD cavity (inflection point—95.7 °C) [50,51], and the other to the breakdown of the macrocycle, at 320.2 °C. The guests show a single mass loss in the temperature range 180–340 °C (EE), 200–360 °C (HD) and 260–500 °C (HN), explained by the degradation of benzene moiety from their structures.

The EE inclusion complex undergoes mass losses in three steps, the first step corresponds to the dehydration of the molecules, the second step to the decomposition of β-cyclodextrin and the last step can be associated with the decomposition of the ester. Regarding the behavior of the HD and HN complexes, the mass loss occurs in two stages. The first stage can also be related to the dehydration of the molecules, as for the second stage, this can be attributed to both, the decomposition of β-cyclodextrin, and the decomposition of the guests. In order to prove the formation of the inclusion complex, the physical mixtures of β-cyclodextrin and guests (EE, HD, HN) were subjected to thermal analysis and the result were compared with those obtained for inclusion complexes. Thus, the first mass loss in the case of the inclusion complexes occurs at 68.5 °C (EEC), 66.7 °C (HDC), and 68.4 °C (HNC), while in the case of the physical mixtures, it occurs at 78.8 °C (EEPM), 78.6 °C (HDPM), and 79.5 °C (HNPM). Moreover, the mass loss corresponding to the degradation of the CD macrocycle occurs at 320.2 °C, and that of the guest’s degradation at 294.5 (EE), 312.8 (HD), 306.7 (HN), as for the complexes, the values were lower (279.1 °C—EEC; 290.2 °C—HDC; 294.9 °C—HNC). These changes in the thermal stability of the host and guest suggest the formation of the inclusion complexes.

**Figure 13:**
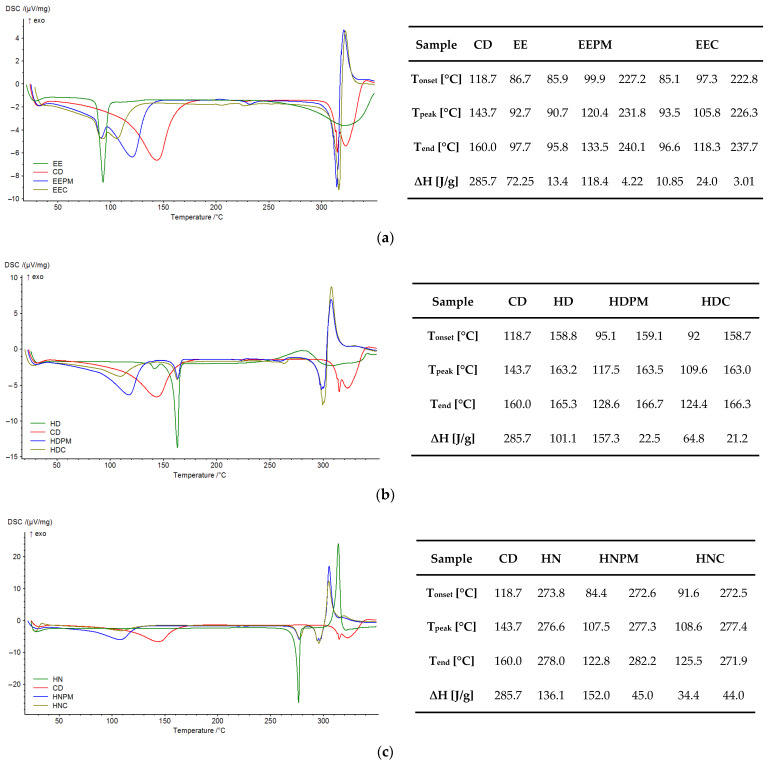
DSC curves and parameters: (**a**) ethyl ester set; (**b**) hydrazide set; (**c**) hydrazone set.

In addition, the results of the DSC analysis (Figure 13) show changes in the enthalpy values corresponding to the thermal phenomena of the two components of the inclusion complexes, proved by the reduction in their peak area in the binary system, in comparison with the physical mixtures. Also, the peaks corresponding to the dehydration and melting/decomposition were shifted to different temperature values in cases of the complexes, proving that the formation of the inclusion complexes altered the thermal degradation property of the two components.

Thus, the ethyl ester (EE) showed a sharp endothermic peak at 92.7 °C, assignable to its melting point [15] and one broad endothermic peak around 322.0 °C, assignable to its decomposition. β-CD has a broad endothermic peak around 143.7 °C, which corresponds to the loss of water molecules from its cavity, and two other endothermic peaks at 315.0 °C and 323.2 °C which can be assigned to the melting and decomposition of the cyclodextrin [52]. The physical mixture (EEPM) and the inclusion complex (EEC) showed the peak of the ethyl ester (90.7 °C and 93.5 °C, respectively) and the endothermic water peak of cyclodextrin (120.4 °C and 105.8 °C, respectively), as well as the peaks around melting point (314.5 °C and 316.4 °C, respectively), and degradation (321.1 °C and 322.6 °C, respectively) of β-CD. Moreover, one broad endothermic peak appears in DSC of both the physical mixture and the complex (231.8 °C and 226.3 °C, respectively), without correspondence in the guest or host, which can likely represent the breaking of non-covalent interactions (hydrophobic interactions, hydrogen bonds) between the guest and β-cyclodextrin, and can be interpreted as a pre-decomposition of the binary compounds [53]. However, when comparing the fusion enthalpy values (ΔH) of the EE melting process, 13.4 J/g (physical mixture) vs. 10.85 J/g (complex), it seems that the guest’s crystalline structure is disrupted more in the inclusion complex than in the physical mixture, suggesting molecular encapsulation or stronger host–guest interactions. An even bigger difference was observed for dehydration enthalpy values (ΔH) of CD, from 118.4 J/g (physical mixture) to 24.0 J/g (complex). Complex formation is driven by the release of high-enthalpy water molecules from the cyclodextrin cavity, which are poorly accommodated and energetically replaced by less polar drug molecules, leading to a more stable host–guest system [54,55]. Smaller changes were observed in case of the hydrazide inclusion complex (HDC), especially on the peak corresponding to the dehydration of the CD, and minor for the hydrazone complex (HNC), suggesting that the inclusion complexes are primarily stabilized by hydrophobic interactions. Also, it is worth noting that the endothermic decomposition of pure components changed to exothermic in the physical mixtures and complexes of EE and HD, maybe due to new molecular interactions that introduce more energetically favorable and heat-releasing degradation pathways, especially in the confined environment of an inclusion complex or through solid-state interactions in physical mixtures [56].

Based on the differences in the X-ray spectra, SEM images, IR spectra, TG and DSC curves between the inclusion complexes and the pure cyclodextrin and guests, the formation of the inclusion complexes was proved.

### 3.3. Antimicrobial Effect

The antibacterial effect of the *N*-(2-bromo-phenyl)-2-hydroxy-benzamide derivatives and their complexes is shown in Table 14. As can be seen, only Gram-positive bacteria were inhibited by the tested compounds, and no inhibition on Gram-negative bacteria was observed at the tested concentrations. Such findings were also remarked in other studies, regarding the evaluation of the antimicrobial activity of some halogenated salicylanilide derivatives [23,57,58,59].

Esters of halogenated salicylanilide with amino acids were evaluated against *Staphylococcus aureus*, methicillin-resistant *Staphylococcus aureus*, *Staphylococcus epidermidis*, *Enterococcus* spp., *Escherichia coli*, and *Klebsiella pneumoniae*. (*S*)-2-(4-Bromophenylcarbamoyl)-5-chlorophenyl 2-acetamido-3-phenylpropanoate and (*S*)-4-chloro-2-(4-(trifluoromethyl)phenyl carbamoyl)phenyl 2-acetamido-3-phenyl propanoate exhibited good antibacterial effect (MICs = 0.98–31.25 μmol/L) against Gram-positive bacteria. Gram-negative bacteria were less susceptible to the action of tested compounds (MICs = 15.62–500 μmol/L) [59].

The antimicrobial activity of some salicylanilide 4-(trifluoromethyl) benzoates was evaluated. Gram-positive bacteria, including MRSA, were inhibited with MICs ≥ 0.49 μmol/L, while among Gram-negative strains, only *E. coli* had a partial susceptibility (MICs ≥ 31.25 μmol/L) [58].

Higher resistance up to 500 μmol/L of Gram-negative species was also observed for salicylanilide diethyl phosphates, meanwhile good activity against Gram-positive bacteria (MICs ≥ 1.95 µmol/L) was obtained [57].

In a previous study conducted in our laboratory, *N*-(2-chlorophenyl)-2-hydroxybenzamide and *N*-(4-chlorophenyl)-2-hydroxybenzamide derivatives were tested against Gram-positive and Gram-negative bacteria. Only the 2-Cl-substituted derivatives showed good activity against Gram-positive bacteria strains (MICs = 0.125–0.5 mg/mL), with no inhibition on the Gram-negative ones. The ethyl/methyl esters were twice as active as their corresponding hydrazides. The activity of the inclusion complex of ethyl 2-(2-((2-chlorophenyl)carbamoyl)phenoxy)acetate in β-CD against the tested bacteria was similar with the activity of the neat ester, even if the amount of the ester in the complex was approximately 4 times smaller [23].

The promising activity of salicylanilide derivatives was proven also by Pauk et al. (2013) [13]. Thus, a series of salicylanilides and derivatives, namely esters of *N*-phenylsalicylamides and 2-hydroxy-*N*-[1-(2-hydroxyphenylamino)-1-oxoalkan-2-yl] benzamides, were synthesized, characterized and evaluated against bacterial and mycobacterial strains. Compared with ampicillin, ciprofloxacin or isoniazid standards, some compounds proved better or similar activity. For example, *N*-(4-bromo-phenyl)-5-chloro-2-hydroxy-benzamide exhibited a 0.76 µmol/L CMI, and its ester, 5-chloro-2-[(4-bromophenyl)carbamoyl]phenyl(2S)-2{[(benzyloxy)carbonyl]amino}-3-methylbutanoate, showed 3.75 µmol/L CMI against *Staphylococcus aureus*. The structure–activity relationships regarding antibacterial activity, proved the importance of R_1_ substitution in the para-position to the carboxamide moiety, together with a lipophilic and electron-withdrawing R_2_ and a bulky R_3_ substituent [13].

In the present research, the minimum inhibitory concentration values of the *N*-(2-bromo-phenyl)-2-hydroxy-benzamide derivatives ranged between 2.5 and 5.0 mg/mL, being 10 times higher when compared with the MICs of the *N*-(2-chlorophenyl)-2-hydroxybenzamide derivatives [23]. It can be concluded that the presence of the bromine instead of chlorine on the benzanilide ring decreased the antimicrobial effect.

This time though, the activity of the ethyl ester (EE) and hydrazide (HD) were equivalent, and the inclusion complexes were similar or twice as active as their corresponding neat derivatives. It should be mentioned that salicylanilide derivatives are found in complex solutions in an amount 3–4 times smaller than in solutions of uncomplexed compounds, because the complexes were obtained using a 1:1 molar ratio.

Thus, the most active compounds were the complexes of the ethyl ester (EEC) and hydrazide (HDC), both of them exhibited an activity of MIC = 2.5 mg/mL against *Streptococcus pneumoniae*. The advantage of complexation was also highlighted in the study of Inoue et al. (2020) where the CD complexes of hinokitiol showed identical or higher activity when compared with neat hinokitiol [60]. The lack of activity for the hydrazone compound (HN) can be explained based on the low solubility of the compound in the culture medium due to its high lipophilicity, which can act as a barrier against the diffusion in the substrate.

### 3.4. In Vitro Anti-Inflammatory Activity

Due to the animal risk management, and ethical concerns, in vivo studies can be replaced, if possible, at least in the case of early studies, by the in vitro evaluation of the biological activity [61].

In the present study, in vitro anti-inflammatory activity was evaluated using the protease inhibition assay, as protease enzyme activity is closely correlated with inflammatory processes. Trypsin is a serine protease enzyme secreted by the pancreas that specifically cleaves peptide bonds at the carboxyl side of lysine and arginine residues. Its activity has been associated with inflammatory responses that can result in tissue damage. As a proteolytic enzyme, trypsin activates immune cells such as eosinophils, which play a key role in the body’s first line of defense and immune response. However, excessive production and release of trypsin can trigger an uncontrolled cascade of events, ultimately contributing to the onset of various diseases [62]. Thus, the evaluation of antiproteolytic effect of compounds represents a good way to predict their anti-inflammatory activity.

When using protease inhibitors, the inhibition of the enzyme activity can be explained based on the formation of an enzyme inhibitor complex, where van der Waals’ and hydrogen bonds [63] or even covalent bonds [18] are formed.

The antiproteolytic assay was performed using selected concentrations of hydroxybenzamide derivatives. Dimethyl sulfoxide (DMSO) was employed as the solvent for compound dissolution, while phosphate-buffered saline (PBS) was used for subsequent dilutions to minimize potential DMSO-induced protein denaturation. At low concentrations, DMSO allows proteins to remain preferentially hydrated in aqueous solutions, thereby preserving their native conformation. Protein denaturation typically occurs only at significantly higher DMSO concentrations [64].

Salicylic acid, sodium diclofenac, acetylsalicylic acid are used as positive controls in different assays to compare the anti-inflammatory efficacy of tested compounds. For example, for trypsin inhibition assay, IC_50_ value of salicylic acid (16.6 ± 0.41 µg/mL) was used to compare the anti-inflammatory potential of aconitine produced by endophytic fungus *Acremonium alternatum* [65], IC_50_ of diclofenac sodium (2.61 mg/mL) was used to compare the anti-inflammatory activity of the extracts from *Gomphrena globosa* L. [66] and for the anti-inflammatory activity of the *Enteromorpha compressa* extracts the reference compound, acetylsalicylic acid (IC_50_ = 4.66 µg/mL) was used [67].

In the present study, the antiproteinase action expressed as the percentage of trypsin inhibition, of both, hydroxybenzamide derivatives and acetylsalicylic acid, used as positive controls, was concentration-dependent.

The IC_50_ value of the acetylsalicylic acid was established to be 0.4051 ± 0.0026 mg/mL and was further compared to the ICs_50_ obtained for hydroxybenzamide derivatives (Table 15).

The IC_50_ values of the title compounds were more than sixfold lower than that of the positive control, indicating their superior efficacy in inhibiting trypsin activity. For uncomplexed compounds, the best inhibition was obtained in the case of ethyl ester (IC_50_ = 0.0436 ± 0.0047 mg/mL), followed by the hydrazone (IC_50_ = 0.0560 ± 0.0109 mg/mL) and hydrazide (IC_50_ = 0.0622 ± 0.0070 mg/mL). The results proved the beneficial role of complexation on the efficiency of the title compounds. The most prominent example in this context is the hydrazide derivative, which demonstrated a fourfold enhancement in biological activity upon complexation (IC_50_ = 0.0592 ± 0.0057 mg/mL), taking into account the 1:1 molar ratio employed during complex synthesis.

Similar results were found for the ICs of fenofibric acid and cyclodextrins, which demonstrate superior anti-inflammatory activity, compared to fenofibric acid alone, and recommend the use of such drug release systems for enhanced pharmacological profile of the active substance [25].

## 4. Conclusions

The present study demonstrated that the investigated compounds, *N*-(2-bromophenyl)-2-hydroxybenzamide derivatives, successfully formed inclusion complexes with β-cyclodextrin, as confirmed through physicochemical characterization. The antimicrobial screening proved that the compounds exhibit inhibitory effects against Gram-positive bacterial strains, with enhanced efficacy observed upon complexation with β-cyclodextrin. Furthermore, the neat derivatives displayed excellent antiproteolytic activity compared to the positive control, which was further potentiated in their complexed forms. Besides the role of molecular encapsulation in modulating in vitro biological activity, the results also highlighted a dose-dependent relationship between the concentration of the *N*-(2-bromophenyl)-2-hydroxybenzamide derivatives and their biological effect. However, comprehensive studies regarding the pharmacological profile are still needed to further consider the studied compounds toward clinical application.

## Figures and Tables

**Table 1:** Calculated HL gaps and the frontier molecular orbitals energies of the investigated compounds (B3LYP/6-311+G).

Test Compounds	E HOMO (a.u.)	E LUMO (a.u.)	HL Gap (eV)
EE	−0.2213	−0.0516	4.616
HD	−0.2280	−0.0574	4.640
HN	−0.2112	−0.0645	3.990

**Table 2:** Steric properties of the investigated compounds.

Test Compounds	Area (Å^2^)	Volume (Å^3^)	Polar Surface Area	Ovality
EE	350.82	328.88	42.909	1.52
HD	325.14	304.76	76.856	1.48
HN	454.06	430.74	70.751	1.65

**Table 3:** Number of hydrogen bond acceptors (HBA) and donors (HBD), the partition coefficient and the polarizability of the studied compounds.

Test Compounds	HBA	HBD	logP	Polarizability
EE	4	1	2.947	66.97
HD	4	3	1.374	64.99
HN	5	3	3.410	75.38

**Table 4:** The final Lamarckian genetic algorithm docked state - best binding affinities of the studied compounds with β-cyclodextrin.

E (kcal/mol)	
Test Compounds	β-Cyclodextrin
EE	−5.11
HD	−5.28
HN	−5.59

**Table 5:** Analysis of the ligand–receptor interactions between the investigated compounds and β-cyclodextrin.

Test Compounds	Interactions with β-Cyclodextrin
EE	Atoms in close contact; 2-bromophenyl moiety appears outside the cyclodextrin, while the salicylic skeleton is placed inside the CD
HD	Atoms in close contact; 2-bromophenyl moiety appears outside the cyclodextrin, together with the NH-NH2 group; the salicylic skeleton is placed inside the CD
HN	Atoms in close contact; both the 2-bromophenyl and salicylic moieties appear outside the CD; benzaldehyde moiety is located within the cavity of the CD

**Table 6:** Complexation energy of the inclusion complexes (B3LYP/6-31G).

Inclusion Complex	ΔE = E_complex_ − E_ligand_ − E_β-CD_ (kcal/mol)
EEC	11.92
HDC	20.71
HNC	−13.80

**Table 7:** Modified characteristic vibrations of guests after complexation.

Characteristic Vibrations (cm^−1^)	EE		HD		HN	
	Free	Complexed	Free	Complexed	Free	Complexed
νNH free/assoc. sec. amide	3325.46	3326.98	3311.80	3317.37	3308.81	3309.01
OH phenolic (overlapped in complex with νOH CD)	-	-	-	-	3421.99	3399.40
νC=O ester	1755.45	1755.49	1685.12	1683.65	1711.32	1711.56
νC=O amide	1653.74	1653.33	1655.64	1654.90	1643.01	1642.84
Sk “1600”	1601.70	1602.70	1599.07	1600.09	1600.63	1600.80
γNH + νCN sec. amide	1535.11	1535.05	1536.65	1537.76	1537.82	1538.50
ν^as^COC aromatic	1233.96	1234.93	1260.90	1261.31	1234.33	1234.90
ν^s^COC aromatic (overlapped in complexes with νCO CD)	1064.31	1079.35	1047.39	1079.07	1065.04	1079.36

**Table 8:** Modified characteristic vibrations of β-cyclodextrin after complexation.

CD Characteristic Vibrations (cm^−1^)	Free	Complexed with EE	Complexed with HD	Complexed with HN
ν[OH]	3400.36	3400.20	3400.34	3399.40
ν[CH_2_]	2928.12	2927.97	2929.07	2925.97
ν[C–C]	1156.72	1157.58	1156.55	1156.01
ν[C–O]	1079.23	1079.35	1079.07	1079.36
ν[O–H]	1029.47	1028.73	1029.06	1028.09

**Table 9:** ^1^H chemical shifts (δ, ppm) corresponding to β-CD protons in the free and complexed state.

^1^H β-CD	H-1	H-2	H-3/H-6 (Overlapped)	H-4	H-5
Free	4.839	3.334	3.638	3.308	3.579
Complexed with HD	4.844	3.338	3.643	3.314	3.584
∆δ	+0.005	+0.005	+0.005	+0.006	+0.005
Complexed with HN	4.844	3.338	3.644	3.313	3.586
∆δ	+0.005	+0.004	+0.006	+0.005	+0.007

**Table 10:** Chemical shift changes corresponding to non-aromatic protons of free and complexed hydrazide.

^1^H HD	Non Aromatic Protons (δ, ppm)			
	OCH_2_CO	NHNH_2_	NHNH_2_	CONHAr
Free	4.903	4.348	9.506	10.476
Complex	4.898	4.350	9.510	10.477
∆δ	−0.005	+0.002	+0.004	+0.001

**Table 11:** Chemical shift changes corresponding to aromatic protons of free and complexed hydrazide.

^1^H HD	Aromatic Protons (δ, ppm)							
	H-3	H-4	H-5	H-6	H-9	H-10	H-11	H-12
Free	7.147	7.570	7.174	8.193	7.704	7.160	7.438	8.013
Complex	7.143	7.568	7.172	8.183	7.702	7.153	7.435	8.005
∆δ	−0.004	−0.002	−0.002	−0.010	−0.002	−0.007	−0.003	−0.008

**Table 12:** Chemical shift changes corresponding to non-aromatic protons of free and complexed hydrazone.

^1^H HN	Non Aromatic Protons (δ, ppm)				
	OCH_2_CO	N=CH	OH	CONHAr	CONHN
Free	5.613	8.283	10.393	10.671	11.727
Complex	5.615	8.278	10.402	10.676	11.733
∆δ	+0.002	−0.005	+0.009	+0.005	+0.006

**Table 13:** Chemical shift changes corresponding to aromatic protons of free and complexed hydrazone.

^1^H HN	Aromatic Protons (δ, ppm)										
	H-3	H-4	H-5	H-6	H-9	H-10	H-11	H-12	H-14	H-16	H-17
Free	7.170	7.572	7.206	8.256	7.693	7.124	7.432	8.059	7.951	7.417	6.874
Complex	7.170	7.572	7.204	8.256	7.693	7.120	7.433	8.060	7.952	7.417	6.870
∆δ	−0.018	0	−0.002	0	0	−0.004	+0.001	+0.001	+0.001	0	−0.004

**Table 14:** Antibacterial activity of tested compounds.

Bacterial Strains	Tested Compounds	Disk Diffusion Method (Inhibition Zones in mm)	MIC (mg/mL, *)
*Streptococcus pneumoniae* ATCC 49619	EE	20	5
	HD	20	5
	HN	7	NO
	EEC	24	2.5
	HDC	23	2.5
	HNC	7	NO
	Levofloxacin 5 µg	20	-
*Staphylococcus aureus* ATCC 25923	EE	20	5
	HD	20	5
	HN	7	NO
	EEC	22	5
	HDC	22	5
	HNC	7	NO
	Gentamicin 10 µg	20	-
*Escherichia coli* ATCC 25922	EE	7	NO
	HD	7	NO
	HN	7	NO
	EEC	7	NO
	HDC	7	NO
	HNC	7	NO
	Gentamicin 10 µg	20	-
*Pseudomonas aeruginosa* ATCC 27853	EE	7	NO
	HD	7	NO
	HN	7	NO
	EEC	7	NO
	HDC	7	NO
	HNC	7	NO
	Gentamicin 10 µg	19	-

* NO—compounds exhibited no activity at tested concentrations.

**Table 15:** Antiproteolytic activity of tested compounds.

Trypsin Inhibition	
Tested Compounds	IC_50_ (mg/mL) *
EE	0.0436 ± 0.0047
HD	0.0622 ± 0.0070
HN	0.0560 ± 0.0109
EEC	0.0671 ± 0.0055
HDC	0.0592 ± 0.0057
HNC	0.0567 ± 0.0075
Acetylsalicylic acid	0.4051 ± 0.0026

* Each value represents the mean ± SD, n = 3.

## Data Availability

The original contributions presented in this study are included in the article/Supplementary Materials. Further inquiries can be directed to the corresponding authors.

## Supplementary Materials

The following supporting information can be downloaded at: https://www.mdpi.com/article/10.3390/pharmaceutics17070869/s1, Figure S1: ^1^H-NMR spectra of β-cyclodextrin (CD); Figure S2: ^1^H-NMR spectra of *N*-(2-bromo-phenyl)-2-hydrazinocarbonylmethoxy-benzamide (hydrazide (HD)); Figure S3: ^1^H-NMR spectra of 2-(5-bromo-2-hydroxy-benzylidene-hydrazinocarbonylmethoxy)-*N*-(2-bromo-phenyl)-benzamide (hydrazone (HN)); Figure S4. ^1^H-NMR spectra of β-cyclodextrin–hydrazide inclusion complex (CHD); Figure S5: ^1^H-NMR spectra of β-cyclodextrin–hydrazone inclusion complex (CHN).
