# Supplementary material for: Antimicrobial and Anti-Inflammatory Activity of N-(2-Bromo-phenyl)-2-hydroxy-benzamide Derivatives and Their Inclusion Complexes
Source: Pharmaceutics. 2025 Jul 2;17(7):869. doi: 10.3390/pharmaceutics17070869 (PMC12300325; doi:10.3390/pharmaceutics17070869)
Supplement: Supplementary file 1 [file pharmaceutics-17-00869-s001.zip › pharmaceutics-3702587-supplementary.pdf]

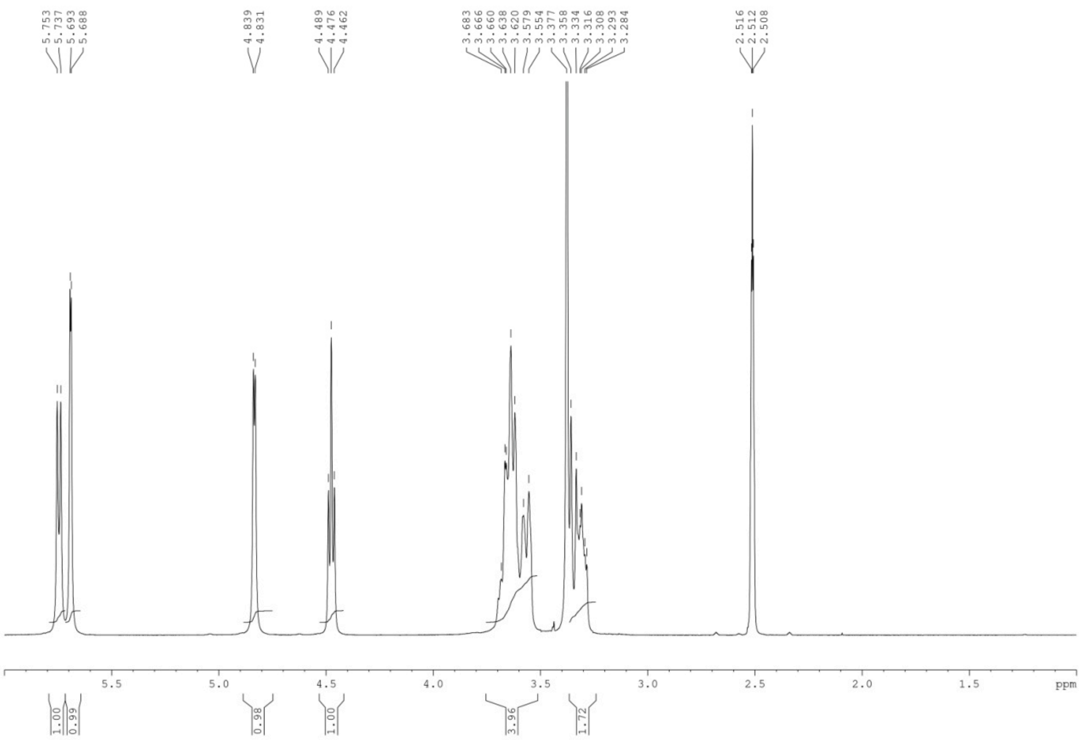

Figure S1. ^{1}H-NMR spectra of β-cyclodextrin (CD)

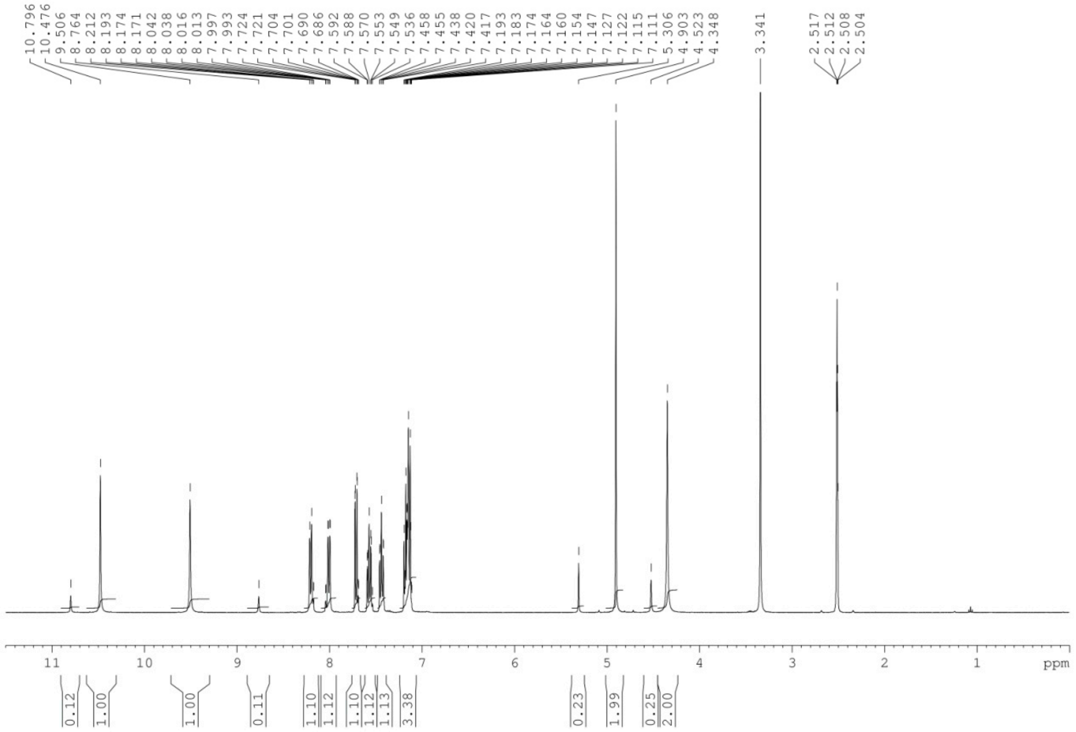

Figure S2. ^{1}H-NMR spectra of *N*-(2-bromo-phenyl)-2-hydrazinocarbonylmethoxy-benzamide (hydrazide (HD))

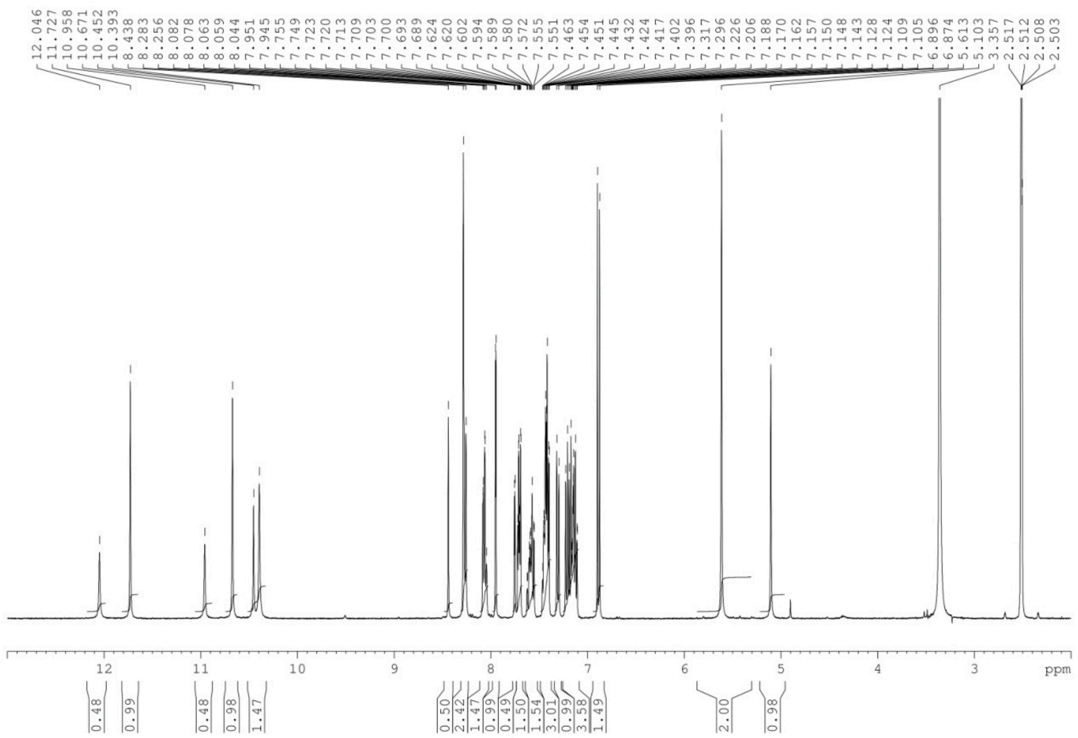

Figure S3. ^{1}H-NMR spectra of 2-(5-bromo-2-hydroxy-benzylidene-hydrazinocarbonylmethoxy)-*N*-(2-bromo-phenyl)-benzamide (hydrazone (HN))

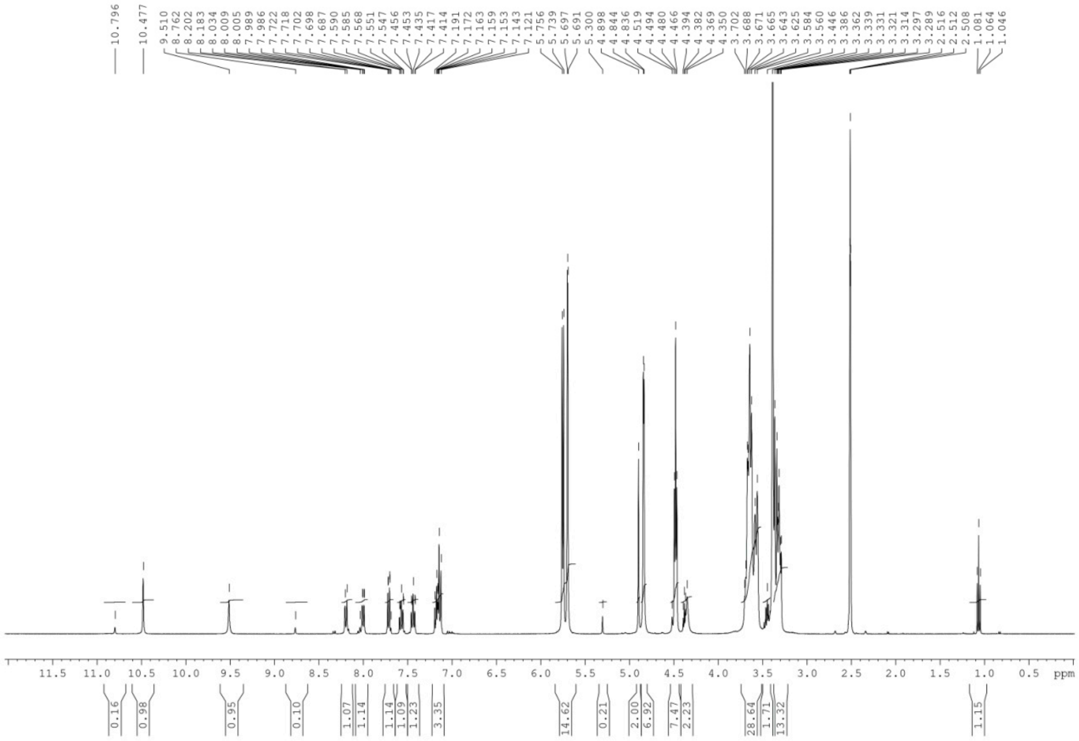

Figure S4. ^{1}H-NMR spectra of β-cyclodextrin – hydrazide inclusion complex (CHD)

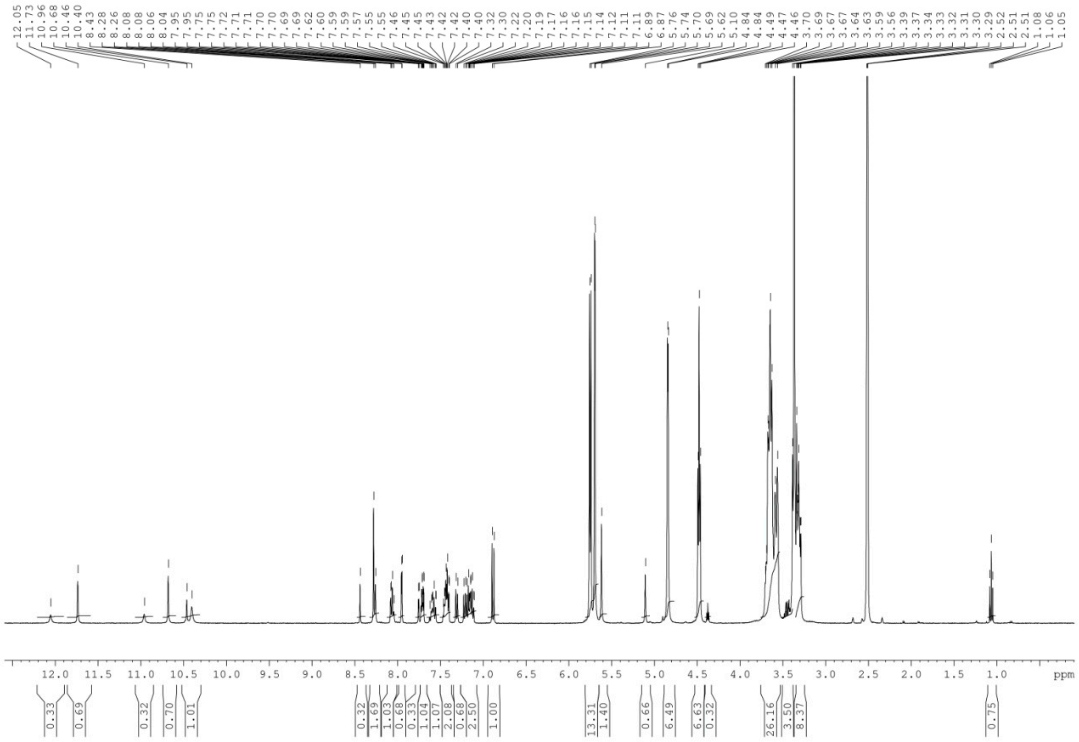

Figure S5. ^{1}H-NMR spectra of β-cyclodextrin – hydrazone inclusion complex (CHN)
